# New insights into the pathophysiology and therapeutic targets of asthma and comorbid chronic rhinosinusitis with or without nasal polyposis

**DOI:** 10.1042/CS20190281

**Published:** 2023-05-18

**Authors:** Ilja Striz, Kornel Golebski, Zuzana Strizova, Stelios Loukides, Petros Bakakos, Nicola A. Hanania, Milos Jesenak, Zuzana Diamant

**Affiliations:** 1 Department of Clinical and Transplant Immunology, Institute for Clinical and Experimental Medicine, Prague, Czech Republic; 2 Institute of Immunology and Microbiology, First Faculty of Medicine, Charles University, Prague, Czech Republic; 3 Subdivision of Allergology and Clinical Immunology, Institute for Postgraduate Education in Medicine, Prague, Czech Republic; 4 Department of Pulmonary Medicine, Amsterdam University Medical Centers, University of Amsterdam, the Netherlands; 5 Institute of Immunology, Second Faculty of Medicine, Charles University and Motol University Hospital, Prague, Czech Republic; 6 Department of Respiratory Medicine, National and Kapodistrian University of Athens, Athens, Greece; 7 First Respiratory Medicine Department, National and Kapodistrian University of Athens, Athens, Greece; 8 Section of Pulmonary and Critical Care Medicine, Baylor College of Medicine, Houston, TX, USA; 9 Department of Pulmonology and Phthisiology, Jessenius Faculty of Medicine in Martin, Comenius University in Bratislava, University Hospital in Martin, Slovakia; 10 Department of Pediatrics, Jessenius Faculty of Medicine in Martin, Comenius University in Bratislava, University Hospital in Martin, Slovakia; 11 Department of Clinical Immunology and Allergology, University Hospital in Martin, Slovakia; 12 Department of Microbiology Immunology and Transplantation, KU Leuven, Catholic University of Leuven, Belgium; 13 Department of Respiratory Medicine and Allergology, Institute for Clinical Science, Skane University Hospital, Lund University, Lund, Sweden; 14 Department of Respiratory Medicine, First Faculty of Medicine, Charles University and Thomayer Hospital, Prague, Czech Republic; 15 Department of Clinical Pharmacy and Pharmacology, University of Groningen, University Medical Center Groningen, Groningen, Netherlands

**Keywords:** airway remodelling, asthma, biologics, chronic rhinosinusitis, precision medicine, type 2 inflammation

## Abstract

Asthma and chronic rhinosinusitis with nasal polyps (CRSwNP) or without (CRSsNP) are chronic respiratory diseases. These two disorders often co-exist based on common anatomical, immunological, histopathological, and pathophysiological basis. Usually, asthma with comorbid CRSwNP is driven by type 2 (T2) inflammation which predisposes to more severe, often intractable, disease.

In the past two decades, innovative technologies and detection techniques in combination with newly introduced targeted therapies helped shape our understanding of the immunological pathways underlying inflammatory airway diseases and to further identify several distinct clinical and inflammatory subsets to enhance the development of more effective personalized treatments. Presently, a number of targeted biologics has shown clinical efficacy in patients with refractory T2 airway inflammation, including anti-IgE (omalizumab), anti-IL-5 (mepolizumab, reslizumab)/anti-IL5R (benralizumab), anti-IL-4R-α (anti-IL-4/IL-13, dupilumab), and anti-TSLP (tezepelumab). In non-type-2 endotypes, no targeted biologics have consistently shown clinical efficacy so far. Presently, multiple therapeutical targets are being explored including cytokines, membrane molecules and intracellular signalling pathways to further expand current treatment options for severe asthma with and without comorbid CRSwNP. In this review, we discuss existing biologics, those under development and share some views on new horizons.

## Introduction

Asthma is a chronic respiratory disease, that is often associated with allergy and/or upper airway involvement and/or other conditions outside of the respiratory tract, such as food allergy and atopic dermatitis [1,2]. The hallmarks of asthma comprise chronic airway inflammation, variable airway narrowing and airway hyperresponsiveness (AHR) to specific and nonspecific triggers as well as structural changes within the airways referred to as airway remodelling [1]. Interestingly, such structural changes were detected within the airways of very young children even before the onset of asthma symptoms [3]. Airway inflammation and structural features may be interrelated in some – though not in all – asthma patients [4–6]. Since the underlying inflammation affects the entire respiratory tract (upper, lower, and small airways), it can be sampled both locally (e.g. nasal brushes, lavage, wash; induced sputum; exhaled breath) and systematically (e.g. peripheral blood and urine) [7].

In the past two decades, our knowledge of the multifaceted nature of asthma and the link to chronic rhinosinusitis has vastly increased [8,9]. So far, two major endotypes have been defined in severe asthma: type 2 ((T2)-high) and non-T2 (or type 2 low) [10].

Type 2 (T2) asthma is presently the best defined endotype, which makes up approximately 50–60% of the asthma population [11,12]. While the majority of patients with T2 asthma usually respond to corticosteroid therapy, a proportion (up to 10%) remains uncontrolled due to unresponsive mechanisms driving their severe asthma [1,13,14]. Targeting these underlying pathways with selective biologics further uncovered the complexity and heterogeneity of severe asthma [15–18]. In parallel, clinically applicable biomarkers (and combinations) helped to identify responders to targeted biologics [19–21]. According to several reports, a substantial proportion of these patients has comorbid chronic rhinosinusitis with nasal polyps (CRSwNP) arising from joint underlying mechanisms [22]. These patients have less favourable disease outcomes, are more difficult to control and are at risk of frequent exacerbations with accelerated lung function decline [23,24]. Although less well-defined in children, the prevalence of severe asthma shows a substantial geographical variability and accounts for approximately 5% of childhood asthma cases. In addition, it is usually characterized by T2 (allergic) inflammation [25].

The definition of non-T2 asthma seems more complex than the one of T2-asthma given the vast heterogeneity of its underlying mechanisms, varying from predominant inflammatory pathways (e.g. airway neutrophilia in the absence of respiratory infections) to the lack of eosinophilic or other inflammation (as in paucigranulocytic asthma), or predominant airway smooth muscle (ASM) dysfunction (characterised by fixed airway narrowing and severe AHR) [6,25]. Pathways and biomarkers underlying non-T2 asthma need further elucidation to allow identification of therapeutic targets, biomarkers and effective targeted treatment options.

In this review, we provide an overview of existing and emerging treatment options for asthma and comorbid CRSwNP, targeting the underlying inflammatory pathways, and discuss our current knowledge and future perspectives.

## Inflammatory pathways and mechanisms underlying asthma and chronic rhinosinusitis

Asthma and chronic rhinosinusitis (CRS) represent frequently occurring, often coexisting diseases, located at the ultimate ends of the respiratory tract and interrelated through joint underlying mechanisms which respond to targeted biologics [16]. Both conditions are highly heterogeneous with a vast interplay among external triggers, microbiome, structural, and inflammatory cells as well as mediator networks contributing to their pathophysiology and clinical presentation [15,26–29].

In the late 1940s, asthma was already identified as a heterogeneous condition and since then a plethora of studies has added accumulating evidence on the stratification of asthmatic patients into different clinical phenotypes and later on, based on the predominant inflammatory sputum cell profile, into inflammatory phenotypes [30].

In the 2000s, asthma was further subcategorised into two major endotypes: Th2 and non-Th2 asthma, based on the presence or absence of (i) CD4+ T-helper cell type 2 (Th2)-driven inflammatory responses (IL-4-, IL-5-, and IL-13-mediated), or Th17-driven responses (IL-17, IL-1β, IL-23), (ii) IgE, and (iii) increased levels of eosinophils, neutrophils, basophils, and mast cells in the airways [11]. More recently, the identification of type 2 innate lymphoid cells (ILC2s) with subsequent studies demonstrating their significant contribution to the pool of hallmark type 2 cytokines, resulted in an update of this terminology to type 2 (T2) and non-type 2 (non-T2) (also known as T2-high or T2-low) asthma, respectively [31].

### T2 asthma

**Figure 1:**
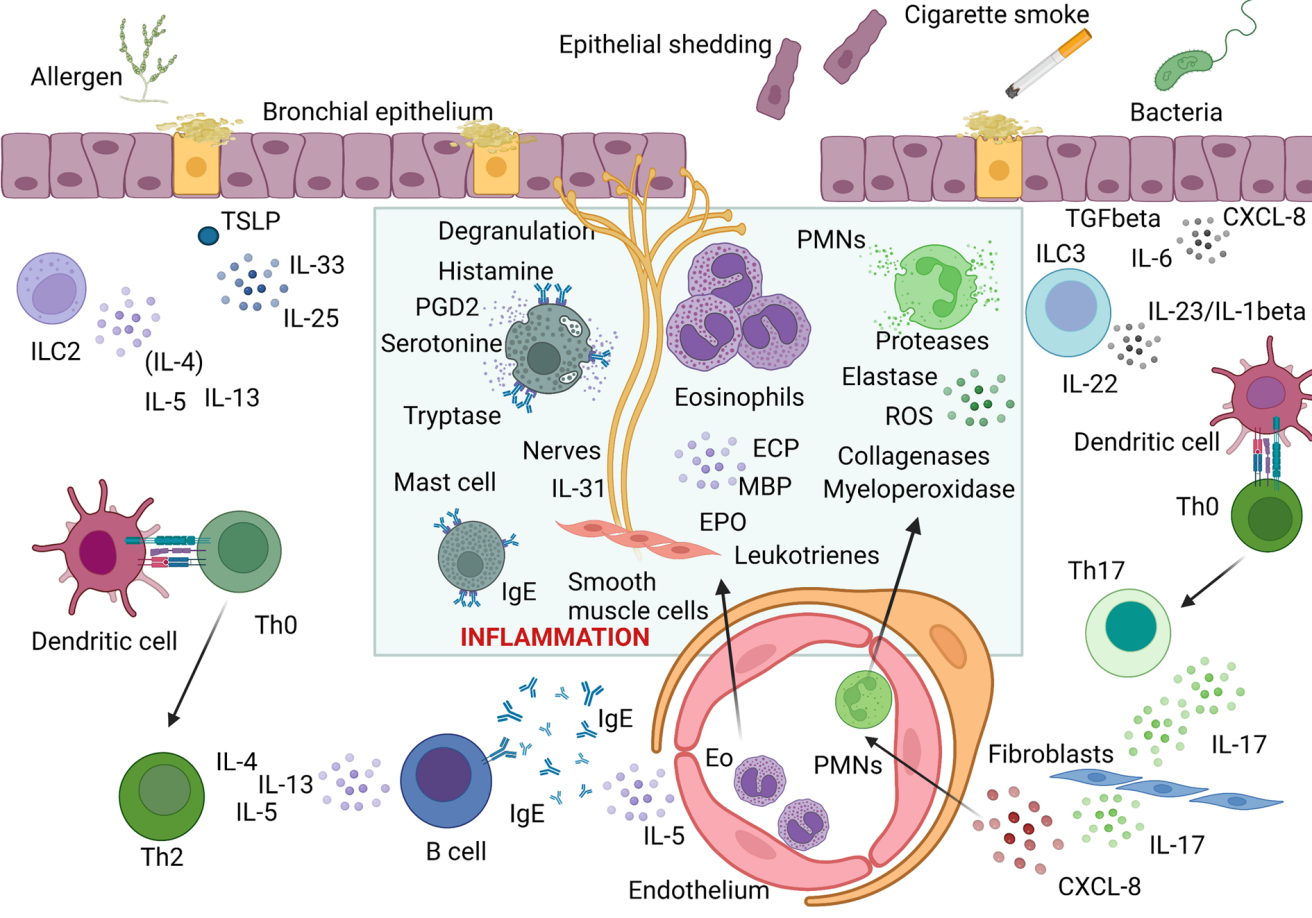
Cellular and cytokine interactions involved in allergic (Type 2) and non-allergic (non-Type 2) asthma. Type 2 immune response is initiated when inhaled allergens trigger epithelial release of thymic stromal lymphopoietin (TSLP), IL-33 and IL-25 which induce type 2 responses via activation of type 2 innate lymphoid cells (ILC2), as well as activation and differentiation of Th2 cells. In this pathway, IL-4 and IL-13 regulate isotype switching in B cells to IgE production while IL-5 regulates eosinophil recruitment and activation. Effector phase of Type 2 immune response is dependent on allergen crosslinking of specific IgE on mast cells and basophils leading to their degranulation and release of histamine, serotonin and other mediators and proinflammatory cytokines. Furthermore, activated eosinophils recruited to the site of allergic inflammation release eosinophilic cationic protein (ECP), major basic protein (MBP) or eosinophilic peroxidase. The mixture of inflammatory mediators and cytokines including IL-4, IL-13, and IL-31 directly affect epithelial cells and induce their shedding, stimulate mucus overproduction, oedema, and bronchoconstriction. In non-type 2 immune response, environmental stimuli including cigarette smoke and microbes lead to a release of neutrophilic chemokine CXCL8, either directly from epithelial cells or fibroblasts via ILC3-mediated IL-17 pathway controlled by epithelial IL-6 and TGF beta. Recruited activated neutrophils release reactive oxygen species (ROS), myeloperoxidase, proteases, elastases and collagenases leading to epithelial cell damage and mucus hyperproduction. Created with BioRender Sofware (Ref.n.WV25BY7AMN).

In T2 asthma, the presence of copious amounts of type 2 cytokines at mucosal sites, as well as intrinsic down-regulation of the expression of claudin-18 and E-cadherins, are linked to the reduced structural integrity of the airway epithelium and the enhanced permeability and responsiveness of the epithelial barrier to exogenous triggers [32–34]. Consequently, airway epithelium in patients with T2 asthma overproduces a broad pallet of pro-inflammatory cytokines and mediators including alarmins (IL-25, IL-33, TSLP), as well as IL-6, IL-8, IL-1α/β, RANTES, or TNF in response to environmental triggers. These mediators create a local pro-inflammatory microenvironment that promotes activation, recruitment and function of other immune cells already residing in the local tissue or upon recruitment from the circulation (Figure 1) [35–37]. Examples of cells activated by epithelium-derived cytokines are dendritic cells (DCs) and ILC2s, which further promote the polarization towards the type 2 immunity by activating T-cells and consequently B-cells. Molecular pathophysiology of T2 asthma is fuelled by activated GATA-3+ CRTH2+ ILC2s as well as Th2 cells that produce substantial amounts of the hallmark type 2 cytokines: IL-4, IL-5, and IL-13. Increased numbers of conventional ILC2s in peripheral blood were found in patients with severe asthma and correlated with disease severity [38]. These ILC2s are likely recruited to the lungs, as increased expression of lung homing receptors was found in blood ILC2s of asthmatic individuals [39,40]. Indeed, a significant increase of IL-5+, IL-13+, and CRTH2+ ILC2s was demonstrated in the sputum of asthmatic patients, 24-48 hours post-allergen challenge coincided with a decrease in blood ILC2s [41]. In line with these findings, PGD2 pathway was found to be up-regulated and correlated to high levels of type 2 inflammatory products in patients with severe uncontrolled T2 asthma [42]. Recently, a novel ILC2 subtype expressing CD45RO was identified in the blood of patients with severe and uncontrolled T2 asthma. CD45RO+ ILC2s are derived from resting CD45RA+ ILC2s upon activation by epithelial alarmins IL-33 and TSLP. Importantly, CD45RO+ ILC2 and their signature cytokines IL-5 and lL-13 were increased in inflamed mucosal tissues and in the circulation of patients suffering from chronic inflammatory diseases mediated by T2 inflammation, including CRSwNP and (late-onset) severe eosinophilic asthma [43,44]. Compared with conventional CD45RA+ ILC2s, these CD45RO+ ILC2s display an unclarified corticosteroid resistance [40,45]. A large body of evidence shows that the impaired activity, expression, and translocation of glucocorticosteroid receptor (GR) alpha plays a significant role in mediating the defective signalling of steroid pathways resulting in corticosteroid resistance. Studies have shown that increased concentrations of type 2 cytokines in the local airway tissues affect GRα translocation and promote corticosteroid resistance, while other studies postulated the defective interaction between GR and transcription factors from the NF-κB and AP-1 families to play a critical role in corticosteroid signalling [46–49]. However, the cause behind the corticosteroid resistance within the already heterogenic population of asthma probably varies between individuals, which constitutes an urgent need for a more personalized medicine approach when treating individual asthma patients.

In contrast with T2 eosinophilic non-atopic asthma which usually manifests at a later age (late-onset), the allergic subset of T2 asthma usually presents at a young age (early-onset). This subtype is characterized by positive allergy skin tests, increased serum total and specific IgE and clinical symptoms upon allergen exposure. Notwithstanding, little is known about the precise roles of cells from the innate and adaptive immune compartments specifically in early-onset allergic asthma as compared with other subsets of T2 non-atopic asthma [50]. In allergic asthma, allergen-specific CD4+ Th2 cells drive inflammatory responses. A differentiation of allergen-specific Th2 cells from naive T cells occurs in draining lymph nodes and is followed by migration to the respiratory tract, most likely through the expression of homing markers, including CCR4 [51–53]. IL-2 signalling was shown to be required for lung homing and retention of long-lived allergen-specific memory Th2 [54]. Adding to the complexity of asthma endotypes, some patients may show both Th17 and T2-mediated airway inflammation, with Th2/ILC2 and Th17/ILC3 profiles and their signature cytokines [55]. Variations in T-cell subtypes in distinct asthma phenotypes including distinct T2 cytokine profiles were demonstrated. In severe asthma, IL-4+/IL-17+ T-cells in BALF expressed higher IL-4 levels compared with IL-4+ T cells. Single-positive IL-4+ T-cells were more frequent in less severe asthma indicating a pathogenic role for IL-4 high cells [56].

Eosinophils, due to their pleiotropic effect on various cell types, are perhaps the most recognized cells in the pathophysiology of T2 asthma. Eosinophils are found at inflamed mucosal sites and upon activation they are capable of producing and releasing numerous pro-inflammatory mediators, including IL-5, IL-13, eotaxins, cysteinyl leukotrienes (CysLTs; including LTC4, LTD4, and LTE4), major basic protein (MBP), eosinophil peroxidase (EPX), eosinophil cationic protein (ECP), and eosinophil-derived neurotoxin (EDN) [57]. Eosinophils significantly contribute to airway tissue remodelling by producing pro-fibrotic factors and consequently activating bronchial fibroblasts [58]. Furthermore, CRSwNP-derived eosinophils enhance IL-5 and IL-13 production by ILC2s, while eosinophil-derived LTD4 was shown to activate naïve ILC2s towards IL-4 secretion [59,60]. In T2 asthma, basophils are recruited to the airway tissues and bronchial walls likely due to increased concentrations of local inflammatory mediators. Activated basophils, as well as mast cells, produce histamine and lipid mediators, prostaglandin D2 (PGD2) and CysLTs [61]. Building on the notion of ILC2 activation by effector cells, one study demonstrated that basophils secreted IL-4 that directly modulated ILC2 towards increased IL-5 production in T2 pathologies [62]. In contrast, a more recent study demonstrated that basophils primed ILC2s to respond to neuron-derived signals necessary to maintain tissue integrity and in this context a dual function of basophils as potent inhibitors of type 2 inflammation was proposed [63].

In children, the eosinophilic phenotype is commonly present which is associated with early-onset disease with atopy, impaired lung function and increased airway hyperresponsiveness [64].

### Non-T2 asthma

Non-T2 asthma (also known as type 2-low-, or non-eosinophilic asthma) is a heterogeneous condition which may comprise several underlying mechanisms including the influx of CD4+ type 1 T helper (Th1) and type 17 T helper (Th17) cells, type 1 and type 3 innate lymphoid cells (ILC1 and ILC3 respectively), neutrophils as well as increased levels of pro-inflammatory mediators in the lung tissues, among others including IL-1ß, IL-6, IL-8, IL-17A/F, IL-22, IFN-γ, and TNF-α. Studies point out that an imbalance in Th17/Treg cells may play a role in corticosteroid-resistant neutrophilic asthma [50,65]. In addition to the dysbalanced immune cell makeup, the pathophysiology of non-T2 asthma may also be mediated by structural abnormalities of the ASM and dysregulated neuronal activation [43,45]. Non-T2 asthma is further subclassified into neutrophilic and paucigranulocytic, depending on the cellular findings in sputum specimens [19,25]. In contrast with T2 asthma, non-T2 is less well defined and largely lacks clinically applicable biomarkers while biologics targeting the presumed underlying mechanisms did not show clinical efficacy so far.

### Chronic rhinosinusitis

CRS is a common chronic inflammatory disorder of the upper airways with an estimated prevalence of approximately 10–15% in the general adult population [66,67]. CRS has a multifactorial aetiology involving several precipitating factors (genetic and environmental) interacting with the upper airway epithelial barrier to trigger inflammatory pathways [68]. Being a frequently occurring comorbid condition in lower airway disorders such as bronchiectasis [69] and asthma (prevalence increasing with asthma severity), physicians are advised to proactively assess both the upper and lower airways in patients as CRS has been associated with worse disease outcome [1,70,71].

Based on endoscopic or imaging findings, CRS comprises two major clinical phenotypes, i.e., with (CRSwNP) or without (CRSsNP) nasal polyps (NP); the latter being approximately twice as prevalent. Although less prevalent, CRSwNP is the most cumbersome phenotype which often coexists in patients with more severe asthma [22,72,73]. Indeed, increasing evidence shows close links at different levels between CRSwNP and asthma, specifically: both conditions are linked through the underlying T2 inflammation, which underscores the united airways concept in this disease subset [72].

An additional clinical subset of CRSwNP and late-onset T2 asthma includes aspirin-exacerbated respiratory disease (AERD) or nonsteroidal anti-inflammatory drugs (NSAIDs) exacerbated respiratory disease (NSAID-ERD). As part of the previously termed ‘Samter's triad’, patients with AERD/NSAID-ERD present with asthma and concomitant CRSwNP with often intractable disease [72,74,75]. The T2-driven pathophysiological mechanism underlying AERD/NSAID-ERD is related to dysregulated arachidonic acid (AA) metabolism and cysteinyl leukotrienes (CysLTs) overproduction [76].

Apart from the aforementioned ‘traditional’ classification, CRS phenotypes are highly heterogeneous and show a substantial geographical with racial variability across inflammatory profiles. For example, in Caucasian populations, CRSwNP most frequently presents with a type 2 inflammatory (eosinophilic) profile, while in Asian populations mixed inflammatory profiles in both CRS clinical phenotypes have been found [72,77]. This aspect should also be taken into consideration in clinical assessment and subsequent treatment plan [78,79].

Based on increased levels of inflammatory proteins and cytokine profiles, CRS (with and without NP) comprises three different endotypes, i.e., T1 characterised by type1 cytokines IFN-γ and TGF-β, T2 by type2 proteins and cytokines IgE and IL-4, IL-5, and IL-13, and T3 by Th17 cytokine IL-17A [77,80,81].

In T2 CRSwNP, eosinophils usually make up the predominant inflammatory cells [82], while ILC2s, B-cells, macrophages, dendritic cells, mast cells and basophils have also been found [28]. Patients with this CRS subtype, may also present with increased levels of FeNO and normal or elevated serum total IgE [83]. However, recent data also point towards the involvement of neutrophils in this subtype [29]. Furthermore, in several studies of CRSsNP, both eosinophils and neutrophils have been demonstrated in upper airway samplings [77].

While NP are commonly found in adults, they are rare in children under the age of 10 years. Furthermore, the presence of NP in children usually indicates underlying systemic diseases, such as cystic fibrosis, primary ciliary dyskinesia or immunodeficiencies. The association with asthma and other allergic/eosinophilic comorbidities is not as clearly expressed in children [84].

## Airway remodelling in asthma and CRS

Structural changes including epithelial abnormalities, subepithelial matrix deposition, ASM cell alterations and mucus hyperproduction contribute to airway remodelling in asthma resulting in small airways disease, non-specific AHR and accelerated lung function decline leading to fixed airflow obstruction [85]. Recent advances in cell biology allowed deeper understanding of the role of diverse non-immune cells contributing to the pathophysiological features of asthma such as AHR [86–88]. Recent data show structural airway changes such as increase in ASM mass and MUC5AC expression in both T2 and non-T2 severe asthma while submucosal glands hyperplasia is associated with T2 intermediate and T2-low asthma [89]. These insights should further move the identification of novel targets for asthma treatment [90,91].

Airway epithelial cells are capable of producing large amounts of cytokines, antimicrobial peptides and multiple proteases, and thus, contribute to the creation of a physical barrier restraining both pathogens and allergens [92,93]. Airway epithelial cells share similar features with innate immune cells, such as the expression of pattern recognition receptors (PRRs) and the secretion of cytokines/chemokines, including IL-6, IL-8, IL-25, IL-33 and CCL20, CCL17 [92,94].

Under specific conditions, such as allergen-mediated epithelial damage, airway epithelial cells lose their protective function, become highly pro-inflammatory and, by promoting airway remodelling, they become crucial players in asthma pathophysiology [95]. Epithelial damage is also characterized by a functional impairment of intercellular junctions caused by the disruption of junctional proteins [92,96]. This phenomenon further increases epithelial permeability and has been demonstrated in all asthma phenotypes [92] as well as in CRSwNP [97]. While DCs mainly impact the differentiation of Th cells into Th1 phenotype, epithelial cells also activate ILC2s and fibroblasts, thus promoting type 2 inflammation and remodelling [98,99]. Eventually, a large proportion of epithelial cells differentiate into mucus-secreting goblet cells [100].

Furthermore, IL-4 and IL-13 released from ILC2s and Th2 cells directly stimulate airway epithelial cells and induce mucus overproduction [101,102]. Dynamic interactions between the innate and adaptive immune cells dictate the polarization of the T-cell immune response [103]. PRRs located on the epithelial cells recognize allergens/infectious antigens and secrete both chemokines and cytokines [104]. As DCs receive cytokine signals from the airway epithelium, the fate of the immune response is decided. Indeed, epithelial cells largely control the differentiation of local DCs [92].

DCs act as antigen presenting cells (APCs) but their actions can vary according to their phenotype [105]. Myeloid and plasmacytoid DCs are the two major phenotypes in human lungs, characterized by either CD11c+/hi, BDCA1+ (CD1c)/BDCA3+ (CD141), and HLA-DR+ for mDCs, or BDCA2CD123+ (IL-3 receptor), CD11c−+ (CD303), BDCA4+ (CD304), HLA-DR+, and ILT7 for pDCs [106]. Antigens are primarily recognized by PRRs of the epithelium and the type of antigenic exposure drives the spectrum of produced cytokine/chemokines, the attraction of DCs and the generation of T-cell subtypes [106,107]. The generally accepted hygiene hypothesis highlights the importance of bacterial stimulation in early childhood, which primes the DCs to induce Th1 polarization and, thus protection against allergies [108]. Different types of allergens, but also non-allergenic proteins, can be discriminated based on diverse surface patterns, such as glycosylation [109]. Other allergens, such as various pollens, may release pollen-associated lipid mediators (PALMs) to trigger the immune response [110]. Glycosylation is essential for recognition by lung epithelial cells and DCs [109]. The most common glycosylation pattern among allergens is mannosylation [85]. While certain antigens are highly immunogenic, some factors were also demonstrated to drive DCs towards tolerogenic phenotype [106,107]. Among these factors, smoking has been shown to alter the functionality of DCs [106].

Another crucial component of airway remodelling represents ASM abnormalities (hypertrophy and hyperplasia) [111]. Structural changes in the ASM cells result in an imbalance between the contractility and relaxation of ASM and subsequently lead to (fixed) airway narrowing and AHR to various stimuli [112]. The increase in the ASM layer is clearly connected to a decreased lung function in asthma. ASM cells are also capable of secreting chemokines, such as CCL11, CXCL10, and CX3CL1, promoting recruitment of mast cells [113]. The release of contractile mediators by mast cells is thought to be particularly responsible for the impairment of ASM contraction [112]. Adhesive and costimulatory molecules expressed on ASM cells further allow interactions with T cells. Several studies showed the capability of ASM cells to migrate within the airway wall [114].

Lung function is also regulated by a network of afferent and efferent nerves [115]. Airway nerves mediate reflexes, as well as ASM contraction [116]. Most airway afferent nerves are unmyelinated C-fibers, however, other fibre types can be activated in the presence of chemical or mechanical factors [116,117]. Efferent fibers consist of parasympathetic, sympathetic, and non-adrenergic non-cholinergic (NANC) nerves regulating different aspects of ASM tone and bronchoconstriction [116,117]. In asthma, both bronchoconstriction and AHR were previously associated with neuronal activity [118]. Therefore, anticholinergics efficiently serve as modulators of the increased cholinergic tone in the airways [119]. Similarly to changes in DC phenotype, also the neuronal activity in asthma may change due to plasticity and remodelling of airway nerves [117]. Neurotrophins are factors that broadly influence the neuronal plasticity and are released by various cell types in the airways, such as neurons, epithelial cells, ASM cells and leukocytes [116]. Neurotrophin expression was shown to be significantly increased in asthma and thus, novel therapeutics have been designed to target the neuronal plasticity [116].

Dysregulation of the DC-epithelium interactions also represents a crucial aspect in CRSwNP pathophysiology [120]. In CRSwNP, defective epithelial barrier leads to increased tissue permeability and cellular damage which promotes chronic (type 2)-inflammation and remodelling [120,121].

Until recently, no truly disease-modifying therapies existed for asthma and/or CRSwNP. However, in terms of airway remodelling, a study including mild asthmatics receiving only SABA showed that three infusions with anti- IL-5 monoclonal antibody mepolizumab reduced the expression of extracellular matrix proteins (tenascin, lumican, and procollagen III) in the bronchial basement membrane. It also reduced TGF-β1 level in BAL fluid and TGF-β1 mRNA expression in eosinophils [122]. Early interventions with (biologic) treatments targeting (and affecting) components of airway remodelling may change this perspective in the future [123]. Obviously, long-term studies with adequate disease outcomes should provide the answer.

## The role of microbiome

Several triggers and external factors (e.g., stress, age, lifestyle, diet, body composition, and medications) can directly or indirectly interfere with our immune system. In this complex interplay, it has become evident that the microbiome plays an important regulatory or even ‘disease driving or modulating’ role [124]. For instance, nutritional products, supplements and medications can alter the composition of the microbiome and thus indirectly affect the immune system [125]. Coinciding with a decreased diversity of beneficial commensals and prevailing pathogens (gut microbial dysbiosis) and a decline in immunity later in life, a potential link with late-onset asthma has been suggested [126]. More recently, other associations between (environmental and endogenous) microbiota and asthma phenotypes have been suggested [127,128].

An increasing number of studies support the existence of the gut-lung axis and the role of gut microbiome alteration in the pathogenesis of chronic inflammatory diseases including asthma and COPD. Only recently, this association has been underscored by data from a large prospective population-based study (FINRISK02; *n* = 7115 adults; follow-up over 15 years) using metagenomic sequencing of stool samples [129]. Particularly, the abundance of *Escherichia, Enterococcus*, *Clostridium*, *Veillonella*, and *Bacteroides fragilis* was found to be associated with a higher incidence of asthma over time [129]. In another study, gut abundance of Clostridium sp. during the first 3 months of life was found to be related to preschool-onset of asthma [130]. Lipopolysaccharide (LPS), an endotoxin originating from the cell wall of Gram-negative bacteria present in the airways and gut of asthmatics, may be responsible for NF-κB dependent inflammation leading to asthma [131]. Also patients with allergic rhinitis display a reduced gut microbial diversity with lower a Firmicutes to Bacteroidetes ratio as compared with healthy controls [132,133]. In CRS, both microbial dysbiosis and *Staphylococcus aureus* colonization may contribute to its pathogenesis [128] while the latter also associates with the occurrence of concomitant asthma [134]. The nasal bacteriome of patients with allergic rhinitis with and without asthma assessed by 16S rRNA high-throughput sequencing was found to be both compositionally and structurally distinct from healthy controls and to differ in metabolic pathways mainly related to degradation and biosynthesis processes [135].

Therapeutic approaches targeting gut microbiome, e.g., with pre- or probiotics or faecal microbial transplantation (FMT) may be beneficial components of asthma management and several studies with tailored approaches are currently ongoing in different patient populations [131]. In this respect, a probiotic preparation was found to increase microbiome diversity in patients with allergic rhinitis with or without concomitant asthma and to decrease their rhinitis symptom score, improved quality of life, reduced the percentage of blood activated eosinophils and basophils and the serum levels of IL-4 and IL-5 [136].

The role of the microbiome in health and disease as well as the effects of new and existing therapeutic modalities, including the long-term application of corticosteroids and biologics, on the composition of the microbiome in the context of disease control and remission is a new area of exploration with the potential to further shape personalized medicine.

## Biomarkers in asthma and CRSwNP

Clinically applicable biomarkers help clinicians to define inflammatory asthma phenotypes and to identify responders to (biologic) treatments [14,137]. Similarly with the one airway concept reflected in asthma and allergic rhinitis, asthma with concomitant CRSwNP also share similar underlying pathways, most often type 2 eosinophilic inflammation [128] although geographical differences in underlying mechanisms exist [138]. Increased eosinophil numbers can almost always be detected in the circulation or in the airway tissues of these patients, yet they are not the key effector cells in all patients. Apart from blood eosinophils, exhaled FeNO and (to a lesser degree) serum periostin levels also serve as surrogate markers of type 2 inflammation, which is being applied to phenotype/endotype both asthma and CRSwNP patients (Table 1).

**Table 1:** Biomarkers in asthma

Biomarker	Characteristics
**T2 asthma biomarkers**	
**Sputum eosinophils (≥3%)**	● Semi-invasive method, available in specialized centers ● Indicative of more intense eosinophilic airway inflammation, poor adherence to inhaled corticosteroids (ICS), poor asthma control and more frequent exacerbations [140,141] ● Highest at night – lowest at midday [142] ● Sputum eosinophil‐guided management of severe asthma leads to a substantial reduction in exacerbations [143] ● Better biomarker in patients on OCS compared with blood eosinophils [144,145]
**Blood eosinophils (>150 or >300 cells/μl)**	● Significant circadian variations during the day (highest at night, lowest at midday) + intrapersonal fluctuations [142] ● Affected by parasitic infections and systemic corticosteroids [7,46,146] ● ≥ 300 cells/μl associated with eosinophilic inflammation and more frequent exacerbations [147,148]
**Fractioned exhaled nitric oxide (FeNO)**	● Easily measurable and reproducible point-of-care biomarker [149,150] ● Does not correlate with sputum eosinophils [151] ● Influenced by ICS use, smoking, atopy, dietary nitrate intake and viral infection [149,152] value >50 ppb in adults often reflects eosinophilic inflammation [151]
**Exhaled volatile organic compounds (VOCs)**	● Composite biomarker not yet validated for clinical practice ● Influenced by medication, microbiome [153,154]
**Total serum IgE**	● Not accurate in atopic and obese patients [155] ● Currently only used for the calculation of omalizumab dose
**Serum periostin**	● Promotes adhesion and migration of epithelial cells, mucus production, eosinophil infiltration and subepithelial fibrosis thus driving airway remodelling [156,157] ● Correlates with blood eosinophils, serum total IgE, eosinophil cationic protein (ECP), and transforming growth factor-β (TGF-β1) [158] ● Superior in predicting fixed airflow obstruction [159]
**Dipeptidyl peptidase‐4 (DPP‐4)**	● Biomarker for predicting the response to anti–IL‐13 treatment [160]
**Prostaglandin D2**	● Derived from the arachidonic acid metabolism [161] ● Biomarker of AERD/NSAID-ERD [161]
**Eosinophil cationic protein (ECP)**	● Increased during asthma exacerbations [162] ● Reduced after treatment [162] ● May be used as a marker for corticosteroid induction [162]
**Eosinophil derived neurotoxin (EDN)**	● High in severe and uncontrolled asthma, especially with persistent airflow limitation [162,163,166] ● Higher serum levels detected at the exacerbation rather than the stable phase of asthma [162,164] ● Promotes the production of matrix metalloproteinase 9 (MMP-9) thus plays a role in airway inflammation as well as in airway remodelling [165]
**Urinary leukotriene E4 (LTE4)**	● The most reliable biomarker for the diagnosis of NSAID-ERD– NSAID-ERD is associated with chronic rhinosinusitis (CRS) with or without nasal polyps [167–169] ● Increased urinary levels were correlated with lung function decline in T2 severe asthma [167] ● uLTE4 is helpful in order to better endotype patients with CRS and to predict disease severity [170]
**Bromotyrosine**	● Urine biomarker ● May predict exacerbations despite the lack of correlation with other more commonly used biomarkers, such as FeNO and sputum eosinophils [173]
**Non-T2 asthma biomarkers**	
**Sputum neutrophilia**	● In the absence of airway infection, indicates a distinct inflammatory phenotype, i.e., neutrophilic asthma. ● Sputum neutrophil counts correlate with the gene expression of sputum IL‐17A and IL‐8 [2] ● Neutrophil extracellular traps (NETs) down-regulate the expression of the tight junction protein of epithelial cells, thus leading to the damage of epithelial cells together with eosinophil degranulation [171]
**Serum calprotectin**	● Correlates with increased sputum neutrophils [172]
**S100A9 (calcium-binding protein A9, calgranulin B)**	● Significantly higher in patients with neutrophilic asthma [173]
**Serum IL‐17**	● Increased in severe compared to milder asthma [174]
**Soluble TNF and IL‐8**	● Increased levels during asthma Exacerbations [175] ● Increased serum levels have been found in severe asthma compared with healthy subjects [174] ● Associated with elevated peripheral blood neutrophils [176]
**NLRP3 and IL‐1β**	● Increased in neutrophilic asthma [177,178]
**Serum chitinase-3-like protein 1 (CHI3L1) and YKL40**	● Both correlate with sputum neutrophils, myeloperoxidase, Il-8, And Il-6 ● Both induce subepithelial fibrosis [179] ● Considered as potential biomarkers of airway remodeling in neutrophilic asthma [179]

In a cohort of patients with severe asthma, the sinonasal mucosal thickness was correlated with levels of systemic (blood eosinophils) and lower airways (sputum eosinophils, FeNO) type 2 biomarkers, indicative of more severe disease [139]. There is now a substantial body of evidence demonstrating that patients with more prominent underlying inflammation usually show a better clinical response to treatment with T2-targeted biologics (Table 1; Table 2) [7,50,140–179].

## Targeted therapy of asthma with or without CRS

**Figure 2:**
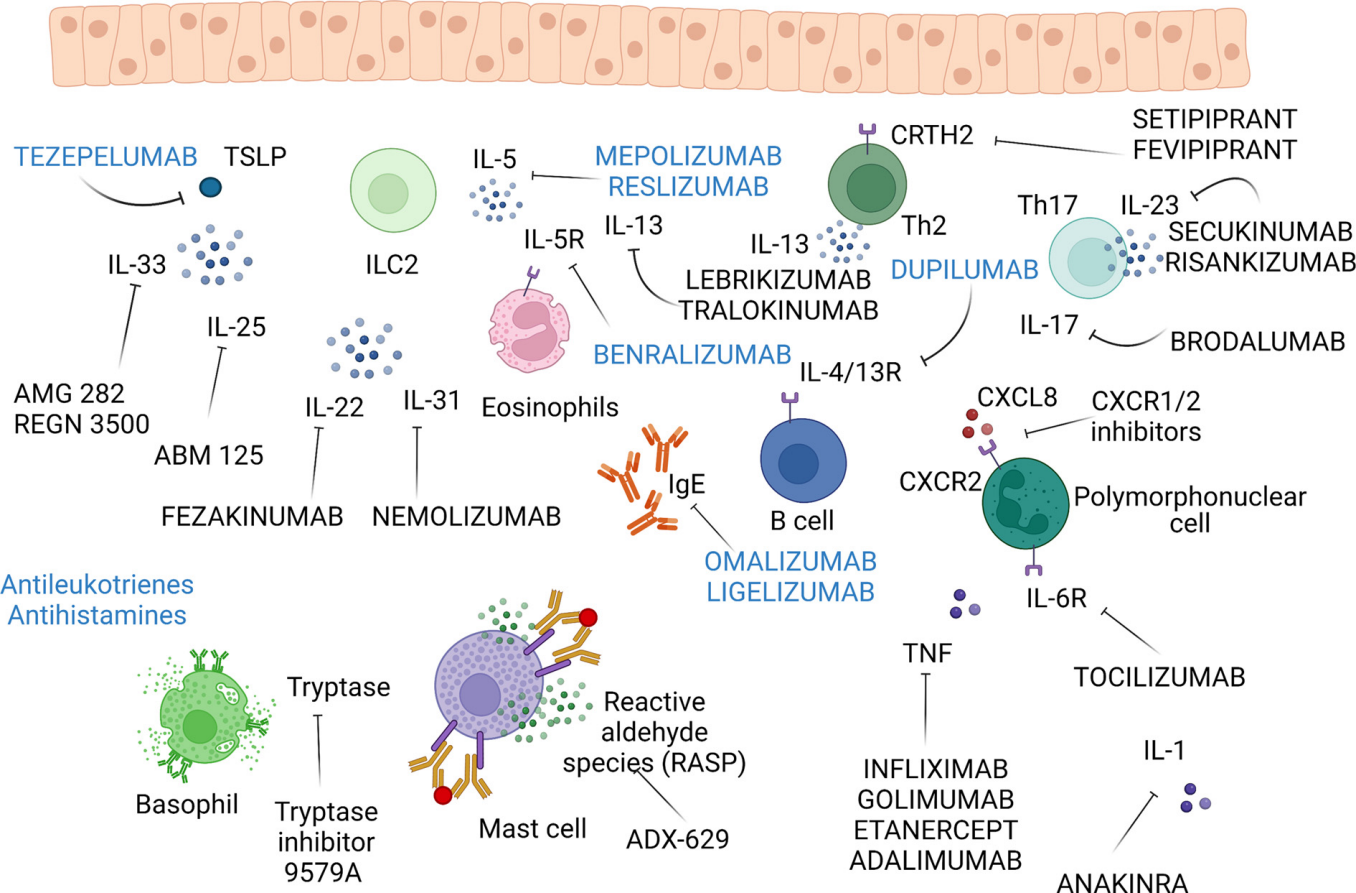
Biologic therapy of bronchial asthma and other allergic disorders. Most of the current monoclonal antibodies and inhibitors target type 2 inflammation (registered biologics in blue letters). Anti – IgE therapy with omalizumab and ligelizumab is already very effective and safe approach to treat patients with difficult asthma. Similarly, anti-IL-5 monoclonal antibodies, particularly mepolizumab, represent an effective therapy of eosinophilic inflammation. Type 2 inflammation can be downregulated also by dupilumab targeting common subunit shared by IL-4 and IL-13 receptors. Recently, tepelezumab directed against thymic stromal lymphopoietin (TSLP) represents another perspective monoclonal antibody modulating type 2 inflammation with another drugs against alarmins IL-33 and IL-25 in clinical studies. Also the inhibitors of CRTH2 (receptor for PDG2) such as fevipiprant are in clinical studies. In non-type 2 inflammation, no biologics are registered for the therapy of asthma with monoclonal antibodies inhibiting IL-17/IL-23 pathway without sufficient clinical effects, so far. In the effector phases of allergic inflammation, antihistamines and leukotrienes are used for decades with emerging inhibitors of tryptase or reactive aldehyde species (RASP) and monoclonal antibodies blocking pro-inflammatory cytokines IL-6, IL-1 and IL-31 in clinical studies. In the case of anti-TNF therapy, the risk potential adverse effects prevail over benefits in asthma therapy. Finally, chemokine receptor inhibitors may represent another tool to block recruitment of immune cells in allergic inflammation. Created with BioRender Sofware (Ref.n.AB25BY6SZW).

Presently, several T2-biologic treatment options exist, blocking the following targets: IgE, IL-5 and IL-5R, IL-4R, and TSLP. A short outline per biologic is provided underneath including the mechanism of action and clinical effectiveness (Table 2, Figure2).

**Table 2:** Targeting immune mechanisms and therapeutic options in T2 and non-T2 inflammation

Target		Approach	Name	Company	Patients	Status/clinical phase
IgE	IgE (Cε3 domain-site involved in binding to FcRI)	Humanized IgG1 MoAb	Omalizumab	Novartis	Asthma Urticaria CRSwNP	Approved
	IgE (Cε3 domain)	Humanized MoAb	Ligelizumab	Novartis	Asthma Urticaria	3
	IgE (M1 segment)	Humanized MoAb	Quilizumab	Genentech	Asthma	2
	IgE (Cε3 domain)	Humanized MoAb	MEDI4212	MedImmune LLC	Asthma	1
	CεmX domain of membrane-bound IgE	Humanized MoAb	FB825	Oneness Biotech Co	Asthma	2
IL-4/IL-13	IL4Ra (subunit common to IL-4 and IL-13 receptors	Human MoAb	Dupilumab	Regeneron/Sanofi	atopic dermatitis asthma CRSwNP EoE	Approved
	IL-4+IL-13 receptors	Recombinant IL-4 variant	Pitrakinra	Aerovance	Atopic dermatitis asthma	2
	IL-4+IL-13	2 MoAbs (VAK694+dectrecumab)	QBX258	Novartis	Asthma	2
	IL-4R	Suman IgG2 MoAb	AMG317	Amgen	Asthma	2
	IL-4	Humanized MoAb	Pascolizumab	GSK	Asthma	3
	IL-4	Human MoAb	VAK694	Novartis	Asthma, pollinosis	2
	IL-4	Soluble recombinant IL-4R	Altrakincept	Amgen	Asthma	Terminated
	IL-13	Humanized MoAb	Lebrikizumab	Lilly	Atopic dermatitis	3
	IL-13	Human MoAb	Tralokinumab	LeoPharma	Atopic dermatitis	Approved
	IL-13	Humanized MoAb	Anrukinzumab	Wyeth	Asthma	2
	IL-13	Humanized MoAb	GSK679586	GSK	Asthma	2
	IL-13	Human MoAb	dectrecumab (QAX576)	Novartis	Eosinophilic esophagitis	2
	IL-13 receptor α1 subunit (IL-13Rα1)	Human MoAb	*ASLAN 004*	Aslan Pharmaceuticals	Eczema	1
IL-5	IL-5	Humanized IgG1 MoAb	Mepolizumab	GSK	Asthma, EGPA HES CRSwNP	Approved
	IL-5	Humanized MoAb	Reslizumab	Teva	Asthma	Approved
	IL-5Ralpha	Humanized MoAb	Benralizumab	AstraZeneca	Asthma	Approved
	IL-5R	Long-acting MoAb	Depemokimab	GSK	Asthma	3
Other molecules regulating T2 inflammation	TSLP	Humanized MoAb	Tezepelumab	AstraZeneca/Amgen	Asthma	Approved
	TSLP	Neutralizing antibody fragment (inhaled form)	CSJ117	Novartis	Asthma	2b
	TSLP receptor	Human MoAb	ASP7266	Upstream Bio	Asthma	1
	IL-33	MoAb	Tozorakimab/MEDI3506	AstraZeneca	Asthma	2
	IL-33	Human MoAb	Itepekimab	Regeneron/Sanofi	Asthma	2
	ST2 (IL-33R)	MoAb	Astegolimab	Hoffmann-La Roche	Asthma	2b
	IL-33R	MoAb	Melrilimab (GSK3772847)	GSK	Healthy subjects	1
	IL-25	MoAb	ABM125	Abeome	Asthma	1/2
	Tryptase tetramers	MoAb	MTPS9579A	Roche/Genetech	Astma	2a
	CRTh2(PGD_2_ receptor)	Antagonist (small molecule)	Fevipiprant QAW039)	Novartis	Asthma	Terminated
	CRTh2(PGD_2_ receptor)	Antagonist (small molecule)	Setipiprant	Actelion	Asthma	2
	IL-9	Humanized MoAb	Enokizumab	AstraZeneca/MedImmune	Asthma	2
	IL-22	Human MoAb	Fezakinumab	Rockefeller University	Atopic dermatitis	2
	IL-31	Humanized MoAb	Nemolizumab	Galderma	Atopic dermatitis	3
	CCR3 (eotaxin receptor)	Inhibitor (small molecule)	GW766944	Glaxo	Asthma	2
	Singlec 8	Humanized MoAb	Lirentelimab	Allakos Inc.	Atopic dermatitis Eosinophilic esophagitis and Duodenitis	2, 3
Potential targets in non T2 inflammation	IL-17	Humanized MoAb	Secukinumab	Novartis	Asthma	Terminated, *
	IL-17RA	Human MoAb	Brodalumab	Amgen	Asthma	Terminated
	C5	Humanized MoAb	Eculizumab	Alexion	Asthma	2
	CXCR2	Receptor antagonist (small molecule)	AZD5069	AstraZeneca	Asthma	2
	CXCR1/2	Receptor antagonist (small molecule)	SCH527123	MSD	Asthma	2
	IL-6	Humanized MoAb	Tocilizumab	Roche	Asthma	Small proof‐of‐concept clinical trial, *
	IL-1	rIL-1 receptor antagonist	Anakinra	Sobi	Asthma	2, *
	IL-1 alpha	Human MoAb	Bermekimab	Janssen	Atopic dermatitis	2b
	Reactive aldehyde species (RASP)	RASP inhibitor (small molecule)	ADX-629	Aldeyra	Asthma	2
	TNF	TNFR:Fc	Etanercept	Sobi	Asthma	2, *
	TNF	Chimeric MoAb	Infliximab	MSD	Asthma	2, *
	TNF	Human MoAb	Golimumab	Centocor	Asthma	2, *
						

*Other indications have been previously approved (within rheumatology; inflammatory bowel disease, psoriasis, autoinflammatory diseases, etc.)

### Targeting IgE

Omalizumab, a recombinant humanized anti-IgE monoclonal antibody, was the first biologic drug aimed at uncontrolled severe allergic asthma, which effectively reduced severe asthma exacerbations, improved asthma symptoms and lung function, and decreased the use of inhaled glucocorticosteroids with a long established (almost 20 years), favourable safety profile. It is approved for patients with allergic asthma older than 6 years [180]. In addition to binding free IgE, omalizumab also downregulates high- and low-affinity Fc receptors for IgE (FcεRI and FcεRII - CD23) expressed on membranes of immune cells, particularly mast cells and eosinophils [181]. More recently, omalizumab also showed efficacy in patients with CRSwNP irrespective of allergy – improving both endoscopic, patient reported and clinical outcomes [182].

Based on the success of this approach, new monoclonal antibodies targeting different epitopes on the IgE molecule emerged. Ligelizumab, a high-affinity IgG1kappa humanized anti-IgE monoclonal antibody was shown to be very potent in IgE binding to the FcεRI, but failed to significantly improve asthma control and exacerbation rates in severe asthma, possibly because of its faster clearance as compared to omalizumab [183]. Quilizumab, a humanized afucosalyted IgG1 monoclonal antibody, directed against the M1 prime segment of membrane-expressed IgE (mIgE), down-regulated serum IgE levels without sufficient clinical effects in patients with inadequately controlled asthma [184] or chronic spontaneous urticaria [185]. MEDI4212, a human IgG1 monoclonal antibody, effectively inhibited binding of IgE to FcεRI and decreased IgE serum concentration *ex vivo* [186]. This antibody was well tolerated and in atopic subjects down-regulated serum IgE levels better than omalizumab; however, this effect appeared transient [187]. A new humanized monoclonal antibody FB825 against CepsilonX domain of the mIgE generated for the depletion of IgE producing B cells is currently evaluated in atopic dermatitis (NCT03758716) and allergic asthma (NCT05008965) without any published data, yet.

### Targeting IL-4R (anti-IL4/IL-13)

The relevance of IL-4 and IL-13 in the pathophysiology of asthma has been extensively addressed throughout various studies and reports [188,189]. Both cytokines are involved in IgE production, as well as airway hyperresponsiveness and airway remodelling [188]. The effects of IL-4 and IL-13 are primarily driven by their interactions with immune cells but also by their binding to specific receptors on bronchial fibroblasts, myofibroblasts, and airway smooth muscle cells [190,191]. Both IL-4 and IL-13 activate the α-subunit of the IL-4 receptor (IL-4Rα) and thus, their mechanism of action can be blocked simultaneously through a therapeutic blockade of the IL-4Rα [192]. However, IL-4 also activates a γC subunit of the IL-4 receptor, whereas IL-13 binds to the IL-13 receptor α1 subunit (IL-13Rα1) [192]. Dupilumab is a fully human anti-IL-4Rα monoclonal antibody approved by both the European Medicines Agency (EMA) and the US Food and Drug Administration (FDA) for the treatment of uncontrolled severe asthma [192]. Clinical efficacy of dupilumab has been demonstrated throughout various studies in patients with T2 (moderate to severe) uncontrolled asthma and mainly reflected in the reductions in exacerbation rates, improvement in asthma control and lung function [193–196].

Following a positive phase IIb study [197], a phase 3 clinical trial (Liberty Asthma Quest) evaluating the efficacy and safety of dupilumab in patients with moderate-to-severe asthma, showed a greater treatment response in patients with higher baseline T2 biomarker levels (i.e. blood eosinophils and FeNO), and thus, supports the use of this biologic in patients with a more prominent T2 inflammation. Similarly, in another phase 3 study (Liberty Asthma Venture), dupilumab treatment allowed reductions in OCS use in patients with corticosteroid-dependent severe asthma while improved clinical outcomes as well as FEV1 [198]. In a pooled patient population with severe CRSwNP with comorbid asthma, dupilumab improved both the upper and lower airway outcome measures as well as HRQoL [199] and is currently considered the leading biologic in patients with CRSwNP with or without concomitant asthma. More recently, dupilumab showed clinical efficacy in children (aged 6–11 years) with uncontrolled moderate-to-severe asthma (VOYAGE study), by significantly reducing asthma exacerbation rates and by improving lung function and asthma control [200]. Presently, dupilumab is indicated for the treatment of severe asthma in patients older than 12 years in Europe and for children above 6 years in the US. In children with uncontrolled atopic dermatitis under 6 years, dupilumab improved Eczema Area and Severity Index (EASI-75) and was well tolerated [201]. Recently, dupilumab gained regulatory approval also for the therapy of eosinophilic esophagitis [202].

Another therapeutic agent targeting both IL-4 and IL-13, pitrakinra, a dual IL-4/IL-13 antagonist, reduced asthma exacerbations only in a subgroup of patients with specific gene polymorphisms of IL-4 receptor [203].

Targeted drugs aiming for selective inhibition of IL-13 include lebrikizumab, tralokinumab, anrukinzumab, decrecumab, GSK679586, and ASLAN 004. Out of these agents, only lebrikizumab and tralokinumab have reached phase 3 clinical trials so far. Lebrikizumab, a humanized monoclonal antibody targeting IL-13, was previously shown to improve asthma symptoms particularly in patients with a high serum IgE level, high blood eosinophil counts, and an increased expression of interleukin-13–related genes in the lung [204]. However, in phase 3 clinical trial in patients with uncontrolled T2 asthma, lebrikizumab did not consistently show a significant reduction in asthma exacerbations [205] and hence, further development for asthma has been discontinued. Tralokinumab, a fully human monoclonal antibody neutralizing IL-13 has proven effectiveness in atopic dermatitis, however, a randomized double-blind clinical trial in patients with corticosteroid-dependent asthma failed to demonstrate oral corticosteroid-sparing effects [206] and hence further development for asthma has been discontinued. Furthermore, a study by Panettieri et al. demonstrated inconsistent efficacy of tralokinumab in the treatment of asthma exacerbation frequency, thus, questioning the role of blocking IL-13 alone in managing severe asthma [207].

Among selective IL-4 inhibitors, pascolizumab, AMG-317 and VAK694, were those to reach phase 2 of clinical development. Pascolizumab and VAK694 are monoclonal antibodies neutralizing IL-4 while AMG 317 is an IL-4 receptor antagonist [208]. Although in clinical trials, pascolizumab was shown to be well tolerated, it failed in phase 2 and hence has been discontinued [209]. VAK694 failed to provide additional benefit when added to subcutaneous immunotherapy regardless of the reduction of allergen specific IL-4 producing cells [210]. Similarly, AMG 317 failed to show clinical efficacy in patients with moderate to severe asthma [211] and trials with soluble recombinant IL-4R, altrakincept, used in a nebulized form, were discontinued in phase 1/2 [212].

### Targeting IL-5

Targeting IL-5 has become a well-established therapy for patients with uncontrolled severe asthma caused predominantly by T2 inflammation with eosinophilia [213]. Mepolizumab, one of the first approved anti-IL-5 monoclonal antibodies, was shown in large placebo-controlled trials to cause a substantial reduction in exacerbations in addition to steroid sparing effect in steroid dependent eosinophilic asthma [214–216]. Moreover, mepolizumab use was not associated with major side effects and thus, became a standard treatment option for severe eosinophilic asthma [217]. A study by Caminati et al. providing real-life evidence on mepolizumab treatment confirmed its favorable safety profile and most importantly, its corticosteroid-sparing effect in daily clinical practice [218]. So far, mepolizumab is the only licensed anti-IL-5 therapy which has also been approved for children over 6 years old with a dosing schedule of 40 mg every 4 weeks. Adolescents older than 12 years receive the same dose (100 mg) as adults. Mepolizumab also significantly increased the number of weeks in remission in patients with eosinophilic granulomatosis with polyangiitis (EGPA) [219], reduced flare up rates in subjects with hypereosinophilic syndrome (HES) [220] and reduced the risk of sinus surgery in patients with severe CRSwNP [221,222].

Similar to mepolizumab, reslizumab targets IL-5 and blocks the subsequent recruitment and activation of eosinophils. In clinical trials, reslizumab was associated with reduction in exacerbations, improvements in lung function, and quality of life [223,224]. The real-world outcomes associated with reslizumab use in patients with severe eosinophilic asthma were demonstrated by Wechsler et al. [225]. who showed an excellent treatment response, while only 3.3% of the patients were classified as non-responders [225]. In patients with CRSwNP, a single intravenous infusion of reslizumab reduced the size of nasal polyps for 4 weeks in half of the patients with nasal IL-5 levels predicting the response, decreased nasal ECP levels persisted for 12 weeks only in responders [226]. Benralizumab, another currently approved anti-IL-5 agent, is directed against the alpha subunit of the IL-5 receptor [227]. It was associated with a reduction of ASM, myofibroblasts, and airway expression of TGF-β1 [84]. In a study by Kavanagh et al., the real-life evidence on benralizumab treatment in severe eosinophilic asthma showed significant improvements in all clinical outcome measures, such as FEV1, Asthma Control Questionnaire (ACQ6) and Mini-Asthma Quality of Life Questionnaire (mAQLQ) [228] scores. This was particularly relevant in patients with a strongly eosinophilic phenotype that were classified as super-responders [228]. Furthermore, benralizumab showed efficacy in patients with severe CRSwNP by decreasing nasal blockage score in a randomized placebo controlled study [229] Depemokimab, a long-acting anti-IL-5 receptor monoclonal antibody, is currently being evaluated in phase III clinical trials in severe uncontrolled asthma with an eosinophilic phenotype (NCT04719832).

### Targeting alarmins

Bronchial epithelium derived cytokines, also called alarmins, include thymic stromal lymphopoietin (TSLP), IL-33, and IL-25, regulate the differentiation of ILC2 and Th2 lymphocytes [230] and represent other potential targets of biologic therapy. TSLP with potent T2-inducing capacity [231] can be effectively blocked by humanized monoclonal antibody tezepelumab by preventing TLSP binding to its receptor. Tezepelumab was recently FDA-approved for the add-on maintenance treatment of adult and pediatric patients with severe asthma a 12 years and older [232], showing a substantial reduction in asthma exacerbation rates irrespective of baseline T2 biomarkers. In a phase 2 (CASCADE) study, tezepelumab reduced inflammatory cells (eosinophils, neutrophils, T cells and mast cells) in bronchial submucosa [233,234]. In the NAVIGATOR (phase 3) study, tezepelumab substantially reduced asthma exacerbation rates, improved asthma control, quality of life as well as lung function in all patients – with better outcomes in those with a T2 inflammatory profile. In the present study, tezepelumab substantially decreased blood eosinophils, serum IgE as well as FeNO, suggestive of its interaction with multiple inflammatory pathways [235]. However, in another phase 3 (SOURCE) asthma study, tezepelumab failed to allow OCS dose reduction in the overall study population, while OCS reduction could be achieved in patients with a T2-profile (baseline blood eosinophils ≥150 cells/μL) [236].

Another anti-TSLP drug, CSJ117, a neutralizing antibody fragment, is currently being tested in an inhaled formulation in patients with severe uncontrolled asthma (NCT04410523). An alternative approach to block TSLP is targeting its receptor. A fully human monoclonal antibody against TSLP receptor (TLPR), ASP7266, is currently being evaluated in pre-clinical studies and in a monkey experimental model completely inhibited induced allergic skin reactions [237].

IL-33 is a member of IL-1 cytokine family potentiating both Th1 and Th2 responses [101]. IL-33 directly activates Th2 lymphocytes, eosinophils, mast cells and basophils [238]. Itepekimab, a human IgG4P anti-IL-33 monoclonal antibody, improved asthma control, quality of life and lung function in patients with moderate-to-severe asthma [239]. Furthermore, tozorakimab, a human IgG1 monoclonal antibody against IL-33 is currently being tested in COPD (NCT05166889). Similarly for IL-25, no data regarding blocking monoclonal antibody, ABM125, have been published, yet. No significant improvement in asthma exacerbations or in lung function were observed with humanized anti-IL-9 monoclonal antibody enokizumab [240].

### Targeting other molecules involved inT2 inflammation

Although initial studies with fevipiprant, an oral antagonist of chemoattractant receptor-homologous molecule on T-helper type-2 cells (CRTH2) serving as a receptor of prostaglandin D2, showed promising efficacy in patients with allergic asthma [241], the phase 3 studies failed to confirm clinical effectiveness in asthma [242] or CRSwNP and concomitant asthma [243]. Similarly, another oral CRTH2 inhibitor, setipiprant, despite showing (modest) efficacy in two allergen challenge studies [244,245], failed to improve symptoms in patients with allergic rhinitis [246].

Inhibition of CCR3 receptor for eotaxin, a chemokine attracting eosinophils, by specific inhibitor GW766994 failed to reduce blood or sputum eosinophilia and to improve lung function in patients with asthma [247].

Nemolizumab, a humanized monoclonal antibody against IL-31 receptor markedly reduced pruritus in patients with atopic dermatitis [248] and down-regulated skin inflammatory biomarkers [249]. This pro-inflammatory cytokine from the IL-6 family might also be a target in related airway diseases.

Lirentelimab (AK002), a humanized, nonfucosylated IgG_1_ monoclonal antibody targeting an anti–sialic acid–binding immunoglobulin-like lectin 8 (Siglec-8) expressed by eosinophils and mast cells has been tested in a Phase 2 study in antihistamine-refractory patients with chronic urticaria leading to improved disease control [250]. There are also ongoing studies with lirentelimab in eosinophilic esophagitis (NCT04322708) and duodenitis (NCT04856891).

### Targeting pathways of non-T2 mechanisms

Compared to T2-biologic therapies, with anti-IgE and anti-IL-5 therapies globally used to treat severe allergic and eosinophilic asthma (+/- CRS), respectively, and with IL-4/13R inhibition being highly effective in atopic dermatitis, CRSwNP +/- severe asthma, targeting non-T2 mechanisms with monoclonal antibodies or inhibitors has been much less explored as well as less successful, so far.

Different approaches have been tested to either down-regulate recruitment of neutrophils to the airways, or inhibit cytokines associated with Th1/Th17 inflammation. CXCR1 and CXCR2 receptors for chemokines attracting preferentially neutrophils, particularly CXCL8/IL-8, CXCL3/Groα or CXCL5/ENA-78, seemed to be natural targets to reduce recruitment of these cells. Treatment with AZD5069, a CXCR2 antagonist, failed to reduce exacerbation rates in patients with uncontrolled persistent asthma [251] despite capability to reduce sputum neutrophils [252]. Similarly, a dual CXCR1/2 inhibitor SCH527123 was found to be safe and reduced sputum neutrophils but similarly failed to show adequate clinical impact in patients with severe asthma [253].

A study with humanized anti-IL-17 monoclonal antibody, secukinumab, in asthmatics (NCT01478360) was terminated requiring changes in study design and human anti-IL-17RA antibody brodalumab failed to show clinical efficacy in severe asthma [254].

Inhibition of the pro-inflammatory cytokine TNF-α has been proven to be very efficacious in multiple chronic diseases, including rheumatoid arthritis and inflammatory bowel diseases, and has also been tested in asthma. Infliximab, a humanized monoclonal antibody, was well-tolerated and decreased exacerbation rates in patients with symptomatic moderate asthma [255]. On the other hand, in moderate-to-severe persistent asthma, etanercept, a soluble TNF-alpha receptor, failed to induce beneficial effects [256]. Following treatment with golimumab, a human anti-TNF monoclonal antibody, no effect on severe exacerbations or lung function were found. Furthermore, adverse events including severe infections and malignancies occurred in golimumab treated subjects [257]. A higher risk of TB reactivation during anti-TNF therapy by infliximab or adalimumab represents another potential obstacle [258]. Hence, the individual risk/benefit assessment should be considered for each asthma patient before using TNF-blockers.

IL-1 is another important pro-inflammatory cytokine with a potential role in bronchial asthma [259]. Anakinra, a recombinant human IL-1 receptor antagonist (IL-1RA) was found to improve airway hyperreactivity and down-regulate T1 and T3 responses in an experimental model of asthma induced by fungi sensitization [260]. The initiated clinical study in asthmatics (NCT04035109) was recently stopped by COVID-19 pandemic (according to ClinicalTrials.gov). Regarding other options for IL-1 blocking, a clinical study with bermekimab, an anti-IL-1α monoclonal antibody in patients with atopic dermatitis (NCT04990440) has been terminated due to low efficacy.

Pro-inflammatory effects of IL-6 may be inhibited by a humanized monoclonal antibody against IL-6 receptor, tocilizumab, which failed to show protection against allergen-induced bronchoconstriction in asthmatic subjects [261].

More recently, blocking of IL-22 by the human monoclonal antibody fezakinumab affected transcription of multiple genes associated with severe neutrophilic asthma and atopic dermatitis [262]. In a randomized, double-blind 2a study, fezakinumab improved both clinical symptoms and molecular disease score in patients with moderate to severe atopic dermatitis [263] .

Among new drugs targeting inflammatory mediators, a clinical study with the inhibitor of reactive aldehyde species, ADX-629, is currently recruiting patients with mild asthma (NCT04728711). Furthermore, MTPS9579A, a monoclonal antibody inhibiting tryptase activity by its dissociation from active tetramers into inactive monomers showed a favourable safety profile [264]; currently, its safety and efficacy are being tested in a clinical trial in more severe asthma [NCT04092582].

In addition to targeting immune mechanisms, antibiotic treatment with macrolides seems to reduce the rate of exacerbations requiring hospitalization and improve symptom scores in severe asthma [265]. Macrolides may be effective also in CRS with low serum IgE suggesting non-T2 inflammation [266]. In patients with severe asthma (often with pronounced airway narrowing) unresponsive to maximal pharmacotherapy, bronchial thermoplasty may improve clinical outcomes [267].

## Emerging approaches for asthma treatment

Multiple other potential therapeutic targets are being explored in experimental models of asthma and associated disorders including cytokines, membrane molecules and intracellular signalling pathways.

One of the cytokines of interest is IL-37, an anti-inflammatory cytokine from the IL-1 family inhibiting the production of T2 cytokines in mononuclear cells stimulated with an allergen. Furthermore, IL-37 down-regulated also IL-1β- and IL-33-induced expression of pro-inflammatory cytokines in cultures of airway epithelial cells [268].

IL-11 is a pleiotropic cytokine which was found to be up-regulated in patients with moderate and severe asthma [269] and, given its role in T2 differentiation, might be another potential therapeutic target.

On the other hand, another cytokine, IL-3 is down-regulated in the mucosa of asthmatic children and intranasal rIL-3 effectively reduced airway eosinophilia and mucus production in a murine model of allergic asthma [270].

Protective effects may also be provided by meteorin β/IL-41 showing anti-inflammatory effects in a murine model of allergic asthma [271].

Airway smooth muscle cells may be targeted by inhibition of stromal-interacting molecule 1 (STIM1) which regulates their proliferation and migration, with induction of multiple asthma-associated proteins and driving of the airway hyperreactivity in a murine model of asthma [272].

Selective inhibitor of NLRP3, OLT1177® (dapansutrile), down-regulated T2 and pro-inflammatory cytokines, caspase-1 activity, reduced lung inflammatory cells and airway hyperreactivity in a model of ovalbumin-induced asthma [273].

Poly (ADP-ribose) polymerase (PARP) is involved in the regulation of multiple genes involved in the pathogenesis of bronchial asthma including lung expression of VCAM-1 [274] and in an experimental model, PAR inhibitor olaparib reduced T2 cytokine release in house dust mite exposed mice [275].

Another approach to targeting immune cell communication is to either block or engage with their membrane molecules. CD200R engagement with CD200-Fc reduced activation, proliferation and production of type 2 cytokines in isolated lung ILC2s and downregulated airway hyperreactivity in a humanized mouse model [276].

Dual inhibition of OX40L and CD30L reduced eosinophilic inflammation and inhibited effector memory Th2 lymphocytes expansion in house dust mite challenged mice [277].

In addition to the already mentioned receptor for PGD2, CRTH2, there are multiple other eicosanoid receptors studied as potential targets of asthma therapy as reviewed recently [278,279].

Inhibition of purinergic receptors regulating the release of alarmins HMGB and IL-33 can attenuate experimental asthma onset and reduce the severity of a rhinovirus-induced asthma exacerbation [280].

Intranasal administration of standardized bacterial lysate OM-85 protected against experimental allergic asthma by multiple mechanisms including effects on airway epithelial cells, regulation of IL-33 and type 2 responses, and by DC tolerogenic reprogramming [281].

In experimental models, inhibitors of phosphoinositide-3-kinase (PI3K) and specifically PI3K-Δ were found to decrease total IgE, and T2 cytokines IL-4, IL-5, and IL-13 together with down-regulation of proinflammatory cytokines TNF-α and IL-1β while having no effect on IL-6 [282]. A dual phosphoinositide 3-kinase (PI3K)γΔ inhibitor AZD8154 is currently tested in an inhalation form in a clinical study [283].

To target anaphylaxis, the fast acting IgE inhibitors based on designed ankyrin repeat protein (DARPin) scaffolds were engineered to neutralize free IgE, dissociate preformed IgE/FcεRI complexes and actively remove prebound IgE from FcεRI on blood basophils [284].

In the future, using of microRNAs (miRNAs) inhibiting translation and upregulating mRNA degradation may be an alternative approach to target specific cells or cytokines involved in allergic inflammation [285]. Intracellular signalling of asthma related pathophysiological changes such as Goblet cell metaplasia may be experimentally inhibited also by antisense oligonucleotides [286].

## Summary

Asthma affects over 350 million people globally, and a substantial proportion of this population has concomitant CRS. While many patients can reach a satisfactory disease control with standard therapies (topical corticosteroids with or without long-acting bronchodilators), many others remain uncontrolled with an increased risk for severe exacerbations and accelerated lung function decline [24] or require systemic corticosteroids imposing serious side effects including what has recently been referred to as ‘people remodelling’, i.e., permanent damage due to corticosteroids [1,287].

An expanding number of biologics targeting T2 asthma (+/- comorbid CRSwNP) has already entered clinical practice showing clinical effectiveness in distinct (partly overlapping) inflammatory phenotypes/endotypes: anti-IgE (omalizumab), anti-IL5 (mepolizumab, reslizumab)/anti-IL5R (benralizumab), anti-IL4Rα (anti-IL4/IL-13; dupilumab), and only recently anti-alarmin TSLP (tezepelumab) [1,288]. However, differences in age indications exist among the available biologics. Presently, omalizumab, mepolizumab and dupilumab may be used in adults and children with asthma aged 6–11 years; tezepelumab is available for adults and children aged 12 years and over, while studies in younger children are ongoing.

Apart from offering targeted treatment options and preventing toxic effects associated with corticosteroid-overuse, these new molecules further helped to unravel underlying mechanisms and to define disease subsets. Based on their respective mechanisms of action, some biologics may achieve disease remission [289] and even show disease-modifying potential in distinct patient populations [123]. These potential aspects (and their persistence) are presently being addressed in a number of clinical studies.

So far, no biologic therapies have shown consistent clinical benefits in non-T2 asthma, although the lack of a (sputum) inflammatory signature may present a significant bias in patients well-controlled by ICS with or without targeted treatments [290]. Hence, a clinically practical approach to non-T2 asthma is best guided by identifying (and subsequent treatment) treatable traits both intrapulmonary, e.g., airway infections, airway narrowing and airway hyperresponsiveness as well as extrapulmonary causes: comorbid conditions, obesity, smoking, occupational exposure, etc [131,291,292]. Better understanding of the underlying pathways on a molecular level may lead to new therapeutic approaches further improving personalized care of patients with asthma and CRS.

## Data Availability

This review manuscript does not contain original data.

## Abbreviations

AA: arachidonic acid
AERD: aspirin-exacerbated respiratory disease
AHR: airway hyperresponsiveness
APC: antigen presenting cell
ASM: airway smooth muscle
BALF: bronchoalveolar lavage fluid
CCL: C-C motif chemokine ligand
CCR3: C-C chemokine receptor type 3
CCR4: CC-chemokine receptor 4
CD: cluster of differentiation
CD200-Fc: CD200 fusion protein
CD200R: CD200 receptor
CD30L: CD30 ligand
CD45RA: cluster of differentiation 45RA
CD45RO: cluster of differentiation 45RO
CRS: chronic rhinosinusitis
CRSsNP: chronic rhinosinusitis without nasal polyps
CRSwNP: chronic rhinosinusitis with nasal polyps
CRTH2: chemoattractant receptor-homologous molecule on T-helper type-2 cells
CRTH2+: prostaglandin D2 receptor 2 positive
CXCL: C-X-C motif chemokine ligand
CysLTs: cysteinyl leukotrienes
DARPin: designed ankyrin repeat protein
DC: dendritic cell
ECP: eosinophil cationic protein
EDN: eosinophil-derived neurotoxin
EMA: European Medicines Agency
EPX: eosinophil peroxidase
FcεRI: High-affinity Fc receptor for IgE
FcεRII: CD23 low-affinity Fc receptor for IgE
FDA: U.S. Food and Drug Administration
FeNO: fractional exhaled nitric oxide
FEV1: forced expiratory volume in 1 second
HLA-DR: human leukocyte antigen-DR
HMGB: high mobility group box
HRQoL: health-related quality of life
IFN-γ: interferon-γ
IgE: immunoglobulin E
IgG4P: immunoglobulin G4P
IL: interleukin
ILCs: innate lymphoid cells
LTC4: leukotriene C4
LTD4: leukotriene D4
LTE4: leukotriene E4
MBP: major basic protein
mDC: myeloid dendritic cell
mIgE: membrane-expressed IgE
miRNAs: microRNA
NCT: ClinicalTrials.gov Identifier (National Clinical Trial)
NLRP3: NOD-like receptor family, pyrin domain-containing protein 3
Non-T2: Non-type 2
NP: nasal polyps
NSAID-ERD: nonsteroidal anti-inflammatory drugs exacerbated respiratory disease
NSAID: nonsteroidal anti-inflammatory drug
OCS: oral corticosteroids
PALM: pollen-associated lipid mediator
PARP: poly (ADP-ribose) polymerase
pDC: plasmacytoid dendritic cell
PGD2: prostaglandin D2
PI3K: Phosphoinositide-3-kinase
PRR: pattern recognition receptor
RANTES: regulated on activation, normal T cell expressed and secreted
Siglec-8: sialic acid–binding immunoglobulin-like lectin 8
SOURCE: Phase 3 study evaluating the safety and efficacy of tezepelumab on OCS dose-reduction in asthma
STIM1: stromal-interacting molecule 1
T1: Type 1
TGF-β: transforming growth factor-β
Th: T helper
Th1: T helper cell type 1
Th17: Type 17 T helper
Th17/Treg: Type 17 T helper/Regulatory T cells
Th2: CD4+ T helper cell type 2
TLPR: TSLP receptor
TNF-α: tumor necrosis factor-α
TSLP: Thymic stromal lymphopoietin
VCAM-1: vascular cell adhesion molecule-1
