# Correction: New insights into the pathophysiology and therapeutic targets of asthma and comorbid chronic rhinosinusitis with or without nasal polyposis

**DOI:** 10.1042/CS-2019-0281C_COR

**Published:** 2023-08-16

**Keywords:** airway remodelling, asthma, biologics, chronic rhinosinusitis, precision medicine, type 2 inflammation

The authors of the original article, *Clin Sci (Lond)* (2023) 137 (9): 727-753, would like to correct their paper. The monoclonal antibody depemokimab described as anti-IL-5R, have been corrected to anti-IL5 in the following statement and in the revised Table 2 provided below.

**Table 2:** Targeting immune mechanisms in T2 and non-T2 inflammation

Target	Approach	Name	Company	Patients	Status/clinical phase
IgE					
IgE (Cε3 domain-site involved in binding to FcRI)	humanized IgG1 MoAb	omalizumab	Novartis	Asthma urticaria CRSwNP	approved
IgE (Cε3 domain)	humanized MoAb	ligelizumab	Novartis	asthma urticaria	3
IgE (M1 segment)	humanized MoAb	quilizumab	Genentech	asthma	2
IgE (Cε3 domain)	humanized MoAb	MEDI4212	MedImmune LLC	Asthma	1
CεmX domain of membrane-bound IgE	humanized MoAb	FB825	Oneness Biotech Co	Asthma	2
IL-4/IL-13					
IL4Ra (subunit common to IL-4 and IL-13 receptors	human MoAb	dupilumab	Regeneron/Sanofi	atopic dermatitis asthma CRSwNP EoE	approved
IL-4+IL-13 receptors	recombinant IL-4 variant	pitrakinra	Aerovance	atopic dermatitis asthma	2
IL-4+IL-13	2 MoAbs (VAK694+dectrecumab)	QBX258	Novartis	asthma	2
IL-4R	suman IgG2 MoAb	AMG317	Amgen	asthma	2
IL-4	humanized MoAb	pascolizumab	GSK	asthma	3
IL-4	human MoAb	VAK694	Novartis	asthma, pollinosis	2
IL-4	soluble recombinant IL-4R	altrakincept	Amgen	asthma	terminated
IL-13	humanized MoAb	lebrikizumab	Lilly	atopic dermatitis	3
IL-13	human MoAb	tralokinumab	LeoPharma	atopic dermatitis	approved
IL-13	humanized MoAb	anrukinzumab	Wyeth	asthma	2
IL-13	humanized MoAb	GSK679586	GSK	asthma	2
IL-13	human MoAb	dectrecumab (QAX576)	Novartis	eosinophilic esophagitis	2
IL-13 receptor α1 subunit (IL-13Rα1)	human MoAb	*ASLAN 004*	Aslan Pharmaceuticals	eczema	1
IL-5					
IL-5	humanized IgG1 MoAb	mepolizumab	GSK	asthma, EGPA HES CRSwNP	approved
IL-5	humanized MoAb	reslizumab	Teva	asthma	approved
IL-5Ralpha	humanized MoAb	benralizumab	AstraZeneca	asthma	approved
IL-5	long acting MoAb	depemokimab	GSK	asthma	3
Other molecules regulating T2 inflammation					
TSLP	humanized MoAb	tezepelumab	AstraZeneca/Amgen	asthma	approved
TSLP	neutralizing antibody fragment (inhaled form)	CSJ117	Novartis	asthma	2b
TSLP receptor	human MoAb	ASP7266	Upstream Bio	asthma	1
IL-33	MoAb	tozorakimab/MEDI3506	AstraZeneca	asthma	2
IL-33	human MoAb	itepekimab	Regeneron/Sanofi	asthma	2
ST2 (IL-33R)	MoAb	astegolimab	Hoffmann-La Roche	asthma	2b
IL-33R	MoAb	melrilimab (GSK3772847)	GSK	healthy subjects	1
IL-25	MoAb	ABM125	Abeome	asthma	1/2
Tryptase tetramers	MoAb	MTPS9579A	Roche/Genetech	astma	2a
CRTh2(PGD_2_ receptor)	antagonist (small molecule)	fevipiprant QAW039)	Novartis	asthma	terminated
CRTh2(PGD_2_ receptor)	antagonist (small molecule)	setipiprant	Actelion	asthma	2
IL-9	humanized MoAb	enokizumab	AstraZeneca/MedImmune	asthma	2
IL-22	human MoAb	fezakinumab	Rockefeller University	atopic dermatitis	2
IL-31	humanized MoAb	nemolizumab	Galderma	atopic dermatitis	3
CCR3 (eotaxin receptor)	inhibitor (small molecule)	GW766944	Glaxo	asthma	2
Singlec 8	humanized MoAb	lirentelimab	Allakos Inc.	atopic dermatitis eosinophilic esophagitis and duodenitis	2, 3
Potential targets in non T2 inflammation					
IL-17	humanized MoAb	secukinumab	Novartis	asthma	terminated, *
IL-17RA	human MoAb	brodalumab	Amgen	asthma	terminated
C5	humanized MoAb	eculizumab	Alexion	asthma	2
CXCR2	receptor antagonist (small molecule)	AZD5069	AstraZeneca	asthma	2
CXCR1/2	receptor antagonist (small molecule)	SCH527123	MSD	asthma	2
IL-6	humanized MoAb	tocilizumab	Roche	asthma	small proof‐of‐concept clinical trial, *
IL-1	rIL-1 receptor antagonist	anakinra	Sobi	asthma	2, *
IL-1 alpha	human MoAb	bermekimab	Janssen	atopic dermatitis	2b
Reactive aldehyde species (RASP)	RASP inhibitor (small molecule)	ADX-629	Aldeyra	asthma	2
TNF	TNFR:Fc	etanercept	Sobi	asthma	2, *
TNF	chimeric MoAb	infliximab	MSD	asthma	2, *
TNF	human MoAb	golimumab	Centocor	asthma	2, *

*) other indications have been previously approved (within rheumatology; inflammatory bowel disease, psoriasis, autoinflammatory diseases, etc)

Furthermore, benralizumab showed efficacy in patients with severe CRSwNP by decreasing nasal blockage score in a randomized placebo controlled study [229] Depemokimab, a long-acting anti-IL-5 monoclonal antibody, is currently being evaluated in phase III clinical trials in severe uncontrolled asthma with an eosinophilic phenotype (NCT04719832).

